# Supplementary material for: Intergroup attitudes and contact between Spanish and immigrant-background adolescents using network analysis
Source: PLoS One. 2022 Aug 5;17(8):e0271376. doi: 10.1371/journal.pone.0271376 (PMC9355234; doi:10.1371/journal.pone.0271376)
Supplement: Attachment — Submitted filename: Plos One Review.docx [file pone.0271376.s001.docx]

The authors present an interesting article that draws on a large sample of Spanish adolescents (an understudied population given the topic) with necessary considerations taken regarding ethics and the surveying of minors. I truly enjoyed reading the article.

Here are some considerations that might help make it even better:

CONTEXT AND PARTICIPANTS

Some more on the context might be relevant. For non-Spanish readers “about 10% of the total Student body during the 2019-2020...” (p. 3) fails to highlight the 2000-2020 increase in the immigrant population at large and in schools within Spain. What are the numbers like in the specific area in Spain where data was collected? Relatedly, where were these public secondary schools located? In larger or smaller cities? Within what Autonomous Community or Autonomous Communities? Were all schools from the same city or county? Were they diverse in their percentage of immigrant population? How many total schools are we talking about? How is this area different or similar to other in Spain. Were the Spanish, Moroccan, Romanian and Ecuadorian groups surveyed similar the population of study? If so, could this be highlighted in a table for example? Also, certain language could use some clarification. For example, I understand what the authors mean by “Roma people” but some context for non-European readers might be helpful.

LITERATURE REVIEW

Overall the citations seem a bit dated and few studies in Spain are cited. There is some high-quality recent (2018-2021) research coming out of nearby Catalonia (Spain) that I think the authors might want to read and integrate. These studies also cite other relevant research you might want to look into, for example:

Bobowik, M., Benet-Martínez, V., & Repke, L. (2021). “United in diversity”: The interplay of social network characteristics and personality in predicting outgroup attitudes. Group Processes & Intergroup Relations, [https://doi.org/10.1177/13684302211002918](https://doi.org/10.1177%2F13684302211002918)

Bobowik, M., Benet‐Martínez, V., & Repke, L. (2021, online first). Ethnocultural diversity of immigrants' personal social networks, bicultural identity integration and global identification. *International Journal of Psychology.* <https://doi.org/10.1002/ijop.12814>

Repke, L., & Benet-Martínez, V. (2018). The (diverse) company you keep: Content and structure of immigrants’ social networks as a window into intercultural relations in Catalonia. *Journal of Cross-Cultural Psychology*, *49*(6), 924-944.

Wilson-Daily, A. E., & Kemmelmeier, M. (2021). Who is on our side? complexities of national identification among native and immigrant youth in Catalonia. *Journal of Youth Studies*, *24*(8), 994-1014.

Wilson-Daily, A. E., Kemmelmeier, M., & Prats, J. (2018). Intergroup contact versus conflict in Catalan high schools: A multilevel analysis of adolescent attitudes toward immigration and diversity. *International Journal of Intercultural Relations*, *64*, 12-28.

Also, a bit dated is the reference to Allport. Athough Pettigrew and Tropp (2006) is mentioned, their building off of and improvement of Allport’s original work is not referenced. Pettigrew and Tropp expanded Allport’s original hypothesis and highlighted necessities for its accomplishment. Nevertheless, this is not highlighted in any way in the article. Furthermore, the authors might be interested in consulting and considering some research from Hewstone and colleagues, such as:

Laurence, J., Schmid, K., Rae, J. R., Hewstone, M. (2019). Prejudice, contact, and threat at the diversity–segregation nexus: A cross-sectional and longitudinal analysis of how ethnic out-group size and segregation interrelate for inter-group relations. Social Forces, 97, 1029–1066. <https://doi.org/10.1093/sf/soy079>

Wölfer, R., Faber, N. S., Hewstone, M. (2015). Social network analysis in the science of groups: Cross-sectional and longitudinal applications for studying intra- and intergroup behavior. Group Dynamics: Theory, Research, and Practice, 19, 45–61. https://doi.org/10.1037/gdn0000021

Wölfer, R., Hewstone, M. (2017). Beyond the dyadic perspective: 10 reasons for using social network analysis in intergroup contact research. British Journal of Social Psychology, 56, 609–617. <https://doi.org/10.1111/bjso.12195>

These should be at least read (if not cited) and perhaps contemplated in regard to study limitations. I see that you do reference a 1993 article of Hewstone’s but I would encourage a more in-depth reading of his group’s more recent work.

ANALYSES

I do not see any control for example for socioeconomic status, given the importance that this plays on intergroup attitudes, the autors might want to comment on this. If this data is available I would find a way to include it in the analyses.

Also, if students are nested in classrooms, which are then nested in schools, and then neighborhoods, a study on intergroup contact might want to contemplate multilevel modeling. Was this data collected (i.e., who is in what classroom/School)? The analysis might benefit from “who am I in contact with in this classroom /school” in *ADDITION* to the self-reported “Quality of contact”.

OTHER, MORE MINOR QUESTIONS OR CONSIDERATIONS

If students did not identify as Spanish, Moroccan, Romanian or Ecuadorian did they not fill out a questionnaire? How were the diferent survey options presented to the students?
