# Supplementary material for: Intergroup attitudes and contact between Spanish and immigrant-background adolescents using network analysis
Source: PLoS One. 2022 Aug 5;17(8):e0271376. doi: 10.1371/journal.pone.0271376 (PMC9355234; doi:10.1371/journal.pone.0271376)
Supplement: Attachment — Submitted filename: Response to Reviewers.docx [file pone.0271376.s003.docx]

**PONE-D-21-14451**

**Intergroup attitudes and contact between Spanish and immigrant-background adolescents using network analysis**

Dear Editor and Reviewers,

Thank you very much for your comments. Your recommendations have been included in the new version. The changes are explained below.

**Reviewer 1 felt that the manuscript has improved greatly, however, s/he is not able to assess whether the data analysis is conducted to the satisfactory level. I also am not sure how your results address the hypotheses. Please assist the readers in revising the part of the results. That is, how are the current results respond to the hypotheses listed on p. 9. Please refrain from using abbreviations, it makes readers very difficult to follow the results.**

We have made some changes in the results section by linking each result to the corresponding hypothesis and eliminating all abbreviations.

In addition, the following clarification has been added regarding the structure of the networks (lines 353-358):

*The nodes corresponding to the stereotypes appear as a structure at the top of the networks in all samples. Similarly, nodes corresponding to positive and negative emotions appear below the stereotype structure. At the bottom of the networks, facilitation and harm nodes appear at both ends and are related to emotions. Finally, the contact quality node appears, also in the lower part of the network, and, in general, related to the facilitation and positive emotions nodes.*

Abbreviations have also been removed in the discussion section.

We hope that this will make the reading of the results clearer and simpler (all these changes have been introduced with change control in the manuscript).

Journal Requirements:

**Please review your reference list to ensure that it is complete and correct. If you have cited papers that have been retracted, please include the rationale for doing so in the manuscript text, or remove these references and replace them with relevant current references. Any changes to the reference list should be mentioned in the rebuttal letter that accompanies your revised manuscript. If you need to cite a retracted article, indicate the article’s retracted status in the References list and also include a citation and full reference for the retraction notice.**

We have checked the list of references to ensure that it is complete and correct. We have no citations for papers that have been retracted.
